# SNAP-25 Polymorphisms in Autism Spectrum Disorder: A Pilot Study towards a Possible Endophenotype

**DOI:** 10.3390/pediatric15040068

**Published:** 2023-12-18

**Authors:** Martina Maria Mensi, Franca Rosa Guerini, Michele Marchesi, Matteo Chiappedi, Elisabetta Bolognesi, Renato Borgatti

**Affiliations:** 1 Child Neuropsychiatry Unit, IRCCS Mondino Foundation, 27100 Pavia, Italy; martina.mensi@mondino.it (M.M.M.); renato.borgatti@mondino.it (R.B.); 2 IRCCS Don Carlo Gnocchi Foundation—ONLUS, 20148 Milan, Italy; fguerini@dongnocchi.it (F.R.G.); ebolognesi@dongnocchi.it (E.B.); 3 Child Neurology and Psychiatry Unit, ASST Pavia, 27029 Vigevano, Italy; michele.marchesi01@universitadipavia.it; 4 Department of Brain and Behavioural Sciences, University of Pavia, 27100 Pavia, Italy

**Keywords:** autism spectrum disorder, SNAP25, endophenotypes, attention

## Abstract

While there is substantial agreement on the diagnostic criteria for autism spectrum disorder, it is also acknowledged that it has a broad range of clinical presentations. This can complicate the diagnostic process and aggravate the choice of the most suitable rehabilitative strategy for each child. Attentional difficulties are among the most frequently reported comorbidities in autism spectrum disorder. We investigated the role of SNAP-25 polymorphisms. Synaptosome-associated protein 25 (SNAP25) is a presynaptic membrane-binding protein; it plays a crucial role in neurotransmission and has already been studied in numerous psychiatric disorders. It was also seen to be associated with hyperactivity in children with autism spectrum disorder. We collected clinical, behavioral and neuropsychological data on 41 children with a diagnosis of autism spectrum disorder, and then genotyped them for five single-nucleotide polymorphisms of SNAP-25. Participants were divided into two groups according to the Autism Diagnostic Observation Schedule (ADOS-2) Severity Score. In the group with the highest severity score, we found significant associations of clinical data with polymorphism rs363050 (A/G): children with the GG genotype had lower total IQ, more severe autistic functioning and more attentional difficulties. Our research could be the starting point for outlining a possible endophenotype among patients with autism spectrum disorder who are clinically characterized by severe autistic functioning and significant attentional difficulties.

## 1. Introduction

According to the DSM 5 [1], autism spectrum disorder is diagnosed in children having persistent deficits in social communication and interaction across multiple contexts and a restricted and/or repetitive pattern of behaviors, interests or activities. Social deficits include problems with social–emotional reciprocity, non-verbal communicative behaviors used for social interaction and understanding and/or developing relationships. Behavior patterns include stereotyped or repetitive movements, stereotyped use of objects, repetitive speech, insistence on sameness with rigid adherence to routines, restricted and fixated interests characterized by abnormal intensity or focus, atypical reactivity to sensory inputs and an unusual interest in sensory aspects of the environment. There is substantial agreement on the diagnosis and on the behavioral aspects that allow the diagnosis to be made: in field trials of the DSM 5 criteria [2], the diagnosis of ASD had the highest kappa statistic compared to other diagnoses applicable to children (0.69, indicating a very good inter-rater agreement). The complexity of ASD lies instead in the relationship between its etiopathogenesis, clinical presentation and care strategies: the different clinical presentations are thought to be the consequence of the differences in the specific pathogenetic pathway leading to ASD in a given subject, and this should guide therapeutic interventions [3]. This is, however, far from possible in real-world everyday practice, given that almost no specific and sensitive biomarker is available, and that (mostly as a consequence of this) no well-designed studies have explored the highly relevant question of “what works for whom” [4].

It is evident in the current scientific literature, but also in clinical practice, that there exist different etiopathogenetic mechanisms underlying a complex phenotype which is determined by some shared traits—key points for the diagnosis—but also by substantial between-subject differences in terms of the specific mix of core symptoms and associated features seen in a specific subject [5]. For instance, a well-accepted (and well-known) dis-tinction is based on cognitive level to differentiate between high- and low-functioning autism [6,7].

As a consequence of this etiopathogenetic heterogeneity, clinical history, most effective interventions and long-term prognoses vary widely, and the choice of the most suitable rehabilitative strategy can be challenging; in some cases, it is a matter of time and patience, but it is hard to determine in advance what can work for whom [8,9].

For this reason, studies outlining specific clinical subtypes (endophenotypes [10]), meaning subpopulations of autistic patients sharing some common traits, can help to target treatment and improve long-term prognosis [11]. Coupled with the definition of clinical profiles, genetics can play a major role in this approach [12,13].

Autism spectrum disorder is associated with several genes, mainly involved in neurobiological development, especially at the synaptic level and in the structural stability of brain cells [14,15].

### 1.1. SNAP-25

The synaptosome-associated protein 25 (SNAP-25) gene encodes a presynaptic membrane-binding protein which is one of the three elements of the SNARE complex, along with Syntaxin-1 and synaptobrevin (also referred to as vesicle-associated membrane protein 2, or VAMP2) [16]. In glutamatergic presynaptic neurons, the SNARE complex plays a crucial role in the exocytosis of vesicles in evoked neurotransmitter release. As an action potential reaches the axon terminal, depolarization events stimulate the opening of voltage-gated calcium channels (VGCCs), allowing for the rapid influx of calcium down its electrochemical gradient to stimulate exocytosis via its binding with synaptotagmin 1 (syt-1). SNAP-25 has been shown to negatively regulate this process in glutamatergic neuronal cells, reducing current density through VGCCs and therefore the amount of calcium binding to synaptotagmin 1 and finally causing a decrease in neuronal glutamatergic exocytosis [17].

### 1.2. SNAP-25 in Neuropsychiatric Disorders

The SNAP-25 gene is located on chromosome 20p11.2. Several studies demonstrated that SNAP-25 single-nucleotide polymorphisms (SNPs) are associated with some psychiatric disorders, including schizophrenia [18] and attention deficit hyperactivity disorder (ADHD) [19]. Specifically, the rs363043 polymorphism was found to be significantly correlated with hyperactivity [20] and the rs363050 polymorphism with low cognitive scores in autistic children [21]. Other authors correlated the rs1051312 polymorphism with an alteration in executive functions in patients with schizophrenia treated with atypical antipsychotics [22]. Executive functions have frequently been considered to be a relevant impairment in subjects with autism spectrum disorder.

Moreover, the axis formed by the regulatory microRNA MiR-130 and SNAP25 has been shown to regulate presynaptic alterations in the anterior cingulate cortex in a murine model in which attentive deficits had been induced by lead exposure [23]. ADHD is also a frequent comorbidity with autism spectrum disorder, and there has been a growing body of research regarding the co-occurrence of these developmental disorders [24]. A number of studies have reported the importance of in-depth clinical and neuropsychological assessment to find tailored treatment options in children [25] but also in adults [26].

### 1.3. Aims of the Study

Our study had two main objectives: (1) to assess the distribution of SNAP-25 polymorphisms in a population of autistic children; and (2) to establish if these polymorphisms have any specific correlation with clinical variables. The possible relevance of these correlations could lead to the possibility of more tailored interventions.

## 2. Materials and Methods

We enrolled 41 children (33 males and 8 females, mean age = 4.4 years; s.d. = 2.4; range: 22–147 months), consecutively referred to the Child Neuropsychiatry Inpatient Unit of the IRCCS Mondino Foundation in Pavia from January 2019 to June 2019. For all of these children, a diagnosis of autism spectrum disorder according to DSM-5 criteria (American Psychiatric Association, 2013) was formulated or confirmed. Exclusion criteria were: (1) the demonstration of a genetic malformation or metabolic disease described in the scientific literature to produce an increased risk of developing autism spectrum disorder, obtained by means of biochemical, genetic or instrumental tests; and (2) parental refusal to provide consent for participation in the study. These criteria lead to excluding 4 children due to criterion 1, while no parents refused to participate in the study.

We collected a full developmental and medical history, together with clinical, behavioral and neuropsychological data on the patients enrolled. Cognitive evaluation was conducted both by clinical assessment and using a standardized, individualized test chosen according to the specific characteristics of the patient.

We also used standardized tools to confirm the diagnosis and to assess the specific clinical profile of the patients (Autism Diagnostic Observation Schedule-Second Edition (ADOS-2) [27] and Autism Diagnostic Interview-Revised [28]).

Both parents of each child completed the Child Behavior Checklist (CBCL) [29] to provide information about behavioral and emotional problems and competencies based on their observation of the children’s behavior.

We also collected a 20 mL peripheral blood sample from each patient. This was processed at the Laboratory of Molecular Medicine and Biotechnology of the IRCCS S. Maria Nascente in Milan. Genomic DNA was isolated by phenol–chloroform extraction as per standard procedures. Each child was genotyped for five single-nucleotide polymorphisms (SNPs) of SNAP-25, described in the scientific literature as potentially relevant for neuropsychiatric disorders in a population genetically comparable to the Italian one: rs363039 (G/A), rs363043 (C/T), rs363050 (A/G), rs3746544 (T/G) and rs1051312 (T/C) [30,31,32]. The first four polymorphisms were analyzed using real-time PCR (Polymerase Chain Reaction) using Taqman SNP Genotyping Assays (Applied Bio-systems) on an ABI PRIMS 7000 Sequence Detection System; C_327976_10, C_2488346_10, C_329097_10 and C_27494002_10 Human Pre-Designed Assays (Applied Biosystem, Waltham, MA, USA) were respectively used. In real-time allelic discrimination, two primer/probe pairs labeled with different reporter dyes (VIC-FAM) and complementary to the target template sequence allow for the genotyping of the two possible variants at the SNP site. One fluorescent dye primer is a perfect match to the wild type (allele 1) and the other fluorescent dye primer is a perfect match to the mutation (allele 2). The rs1051312 (T/C) polymorphism was typed through RFLP (restriction fragment length polymorphism), using Ddel digestion as previously described [20].

We used the results obtained from genotyping to subdivide the study population in order to assess between-group differences. These differences were statistically tested using analysis of variance (ANOVA) and Student’s *t*-test for continuous, normally distributed variables; non-parametric equivalents were used for non-normally distributed variables. Chi-square analysis was used to verify if SNAP-25 SNP genotype distribution among children enrolled in the study was in Hardy–Weinberg equilibrium (HWE) and to evaluate possible skewing of genetic distribution. Statistical analysis was conducted using IBM SPSS 21 (Armonk, New York, NY, USA). Results were considered statistically significant when *p*-value was <0.05.

This study was conducted in accordance with the Declaration of Helsinki; all parents or legal guardians provided informed consent to confirm their willingness to participate. This study was approved by the Ethical Committee of the Don Carlo Gnocchi ONLUS Foundation (Milan, Germany) and of the San Matteo Hospital (Pavia, Germany; number 20170034734)).

## 3. Results

Among the enrolled subjects, we found a clear prevalence of children of Caucasian ethnicity (96.2%) and whose parents spoke Italian as their first language (88.8%). Just under half of the patients (46.3%) had a family history of neurological or psychiatric disorders in the maternal or paternal line or in the siblings (the latter alone reaching a smaller percentage, 17.1%). A regular pregnancy was reported in the majority (69.3%) of cases, ending with a full-term (78%) eutocic (65.9%) birth.

The vast majority of the patients (85.4%) had a regular motor development and the mean age of independent walking was 14.9 months (s.d. 3.6); in contrast, in many of them (63.4%), a delay in language development was evident at 24 months. Parents were able to see that something was not going as expected in their children by the age of 24.6 months (s.d. 9.6).

At the time of inclusion, 73.2% were already following a rehabilitation program, consisting in psychomotor therapy in 60% of the cases, psychomotor and speech therapy in 26.7% and behavioral therapy (ABA) in 13.3%. Only 7.3% of patients were also being treated pharmacologically, in all cases with risperidone.

We considered the ADOS-2 our main discriminating assessment, since we referred to its comparison score to divide participants into two groups, the autism group and the autism spectrum group, consisting of children presenting with a greater (the former) and a reduced (the latter) intensity of the core symptoms. Twenty-six patients (seven females, nineteen males; mean age = 3.6; s.d. = 1.5) were assigned to the autism group, while fifteen (one female, fourteen males; mean age = 5.6; s.d. = 3.1) to the autism spectrum one.

The genotype distribution resulted in HWE and no significant differences were observed between the two studied groups.

Correlation with clinical data was performed for all polymorphisms, but significant data emerged only for rs363050 (A/G).

We found significant associations of some clinical data with polymorphism rs363050 (A/G) only in the autism group (see Table 1 for details), specifically concerning the following:

- IQ values: children with a homozygous genotype GG had a significantly lower total IQ (mean = 45.0; s.d. = 17.7) than those with genotype AA (mean = 66.4; s.d. = 14.9; *p* = 0.04);
- ADOS-2 comparison score: children with genotype GG had significantly higher comparison scores (mean = 9.4; s.d. = 0.8) than those with genotype AG (mean = 7.5; s.d. = 1.3; *p* = 0.01) and those with genotype AA (mean = 7.8; s.d. = 1.6; *p* = 0.08);
- ADI-R(C) score, which expresses restricted and fixated interests, was found to be higher in children with genotype GG (mean = 7.0; s.d. = 1.5) compared both to those with genotype AG (mean = 4.4; s.d. = 1.5; *p* = 0.03) and those with genotype AA (mean = 4.0; s.d. = 1.2; *p* = 0.05);
- subscale VI of the CBCL questionnaire, indicative of attention difficulties, in which autistic children with genotype GG showed significantly higher scores than those with genotype AG and AA (fathers: *p* < 0.01; mothers: *p* = 0.028).

## 4. Discussion

The heterogeneity of the autism spectrum is widely acknowledged today, but how to approach it remains controversial [3,4]. Rather than treating it as an obstacle in the search for a prototypical autism pattern [4], this polymorphic clinical presentation can be an occasion to really adapt our clinical interventions to the patient, looking at the individual person rather than at the diagnostic category. In our sample, we had a significant number of preterm births (22%). All of them were “late preterm”; this is a factor that could reduce the generalizability of our findings. The same can be said for the significant number of births which were not eutocic (34.1%); these caesarean births were most often due to breech presentations (26.8%) and sometimes to maternal request (7.3%). In all subjects, fetal conditions at birth were normal according to Apgar scores (none of our subjects had a score below 8) and no sign or symptom of neurological sequelae was detected during evaluation.

There is an area of overlap between the diagnoses of autism spectrum disorder and Attention Deficit/Hyperactivity Disorder (ADHD) [33]. A search for specific clinical subtypes could therefore lead towards a better understanding of the most suitable therapeutic and rehabilitative strategies for each child’s difficulties [34], rather than stemming from an urge for a complete classification or a definitive interpretation of the origin of the disorder.

The definition of an endophenotype could also lead to optimizing the costs generated by interventions in ASD subjects: a recent study conducted in Europe estimated an average cost of health-related services in 2018 to be EUR 1210, while indirect social costs were even higher, reaching on average EUR 1624 [35]. The extremely high standard deviation reported by the authors (EUR 2160 for health-related services, EUR 1317 euros for indirect social costs) can hardly be seen only as a consequence of the different health system organization models. It more probably also reflects an attempt to differentiate treatment strategies, possibly following clinical evaluation, showing significant differences in the specific functioning of these patients.

It is with this idea in mind that we approached the problem of the clinical overlap between autism spectrum disorder and attention deficit/hyperactivity disorder. From a clinical point of view, in fact, impulsivity, inattention and hyperactivity are often present in individuals with autism spectrum disorder, and have a strong intersection with non-social autistic traits, such as repetitive behavior [36]. Interestingly, Sesso et al. reported an increase in impairment in subjects with ADHD who also had ASD [25]; these findings, obtained from a retrospective analysis of youths diagnosed with ADHD, are, however, in line with our evidence of more severe impairment.

Our work highlighted that some polymorphisms of the SNAP-25 gene could have a specific distribution in the ASD population, specifically in subjects showing a greater intensity of symptoms. In this group, children with the rs363050 (GG) genotype had lower total IQ, more restricted and fixated interests, and more attention difficulties. Of note, a very recent paper stated that SNAP-25 “may represent a susceptibility gene for autism in the Han Chinese population”, although it has to be acknowledged that these authors studied SNPs different from those evaluated in the present paper and in a genetically different population [37]. It would be helpful to conduct basic research to test the effect of these polymorphisms, e.g., in animal models, to better understand their effect in humans.

The limitations of our study include the relatively small sample recruited and the cross-sectional design, which prevents us from understanding the efficacy of different therapeutic and rehabilitative approaches in relation to SNAP-25 polymorphisms. Moreover, patients were all recruited in a third-level center, a fact that could have led to selection bias. We are also aware that, given genetic heterogeneity beyond ASD, the SNPs studied in our paper should be added to larger studies investing multiple candidate genetic factors at the same time in sufficiently large samples. This could also allow us to compare subgroups (e.g., with or without family history of neuropsychiatric disorders, or Caucasian vs. non-Caucasian).

Our research, however, could be a starting point for outlining a possible endophenotype among patients with autism spectrum disorder who are clinically characterized by severe autistic functioning and significant attentional difficulties. This could help to differentiate these subjects in terms of the most helpful interventions, both from a pharmacological and a rehabilitative perspective.

## Figures and Tables

**Table 1:** Values of different parameters in the autism group according to rs363050 (A/G) polymorphism. Data are given as means (s.d.).

	A/A	A/G	G/G
IQ	66.4 (14.9)	59.62 (17.3)	45 (17.7)
ADOS-2 comparison score	7.8 (1.6)	7.5 (1.3)	9.4 (0.8)
ADI-R (C)	4 (1.2)	4.4 (1.5)	7 (1.5)
CBCL mother– subscale VI	60 (5.9)	57.25 (5.6)	65.8 (4.6)
CBCL father– subscale VI	57.8 (5.9)	56.9 (6.4)	70.6 (7.5)

## Data Availability

Raw data are available from the corresponding author upon reasonable request.
